# The formyl peptide receptor agonist Ac2-26 alleviates neuroinflammation in a mouse model of pneumococcal meningitis

**DOI:** 10.1186/s12974-020-02006-w

**Published:** 2020-10-29

**Authors:** Marvin Rüger, Eugenia Kipp, Nadine Schubert, Nicole Schröder, Thomas Pufe, Matthias B. Stope, Markus Kipp, Christian Blume, Simone C. Tauber, Lars-Ove Brandenburg

**Affiliations:** 1 Institute of Anatomy, Rostock University Medical Center, Gertrudenstrasse 9, 18057 Rostock, Germany; 2 Department of Anatomy and Cell Biology, RWTH Aachen University, Aachen, Germany; 3 Department of Urology, University Medicine Greifswald, Greifswald, Germany; 4 Department of Gynecology and Obstetrics, University Hospital Bonn, Bonn, Germany; 5 Center for Transdisciplinary Neurosciences Rostock (CTNR), Rostock University Medical Center, Gelsheimer Strasse 20, 18147 Rostock, Germany; 6 Department of Neurosurgery, RWTH Aachen University, Pauwelsstrasse 30, 52074 Aachen, Germany; 7 Department of Neurology, RWTH University Hospital Aachen, Aachen, Germany

**Keywords:** Bacterial meningitis, Formyl peptide receptor, Glial cell, Innate immunity, *Streptococcus pneumoniae*, Annexin A1

## Abstract

**Background:**

Bacterial meningitis is still a cause of severe neurological disability. The brain is protected from penetrating pathogens by the blood-brain barrier and the innate immune system. The invading pathogens are recognized by pattern recognition receptors including the G-protein-coupled formyl peptide receptors (FPRs), which are expressed by immune cells of the central nervous system. FPRs show a broad spectrum of ligands, including pro- and anti-inflammatory ones. Here, we investigated the effects of the annexin A1 mimetic peptide Ac2-26 in a mouse model of pneumococcal meningitis.

**Methods:**

Wildtype (WT) and *Fpr1*- and *Fpr2*-deficient mice were intrathecally infected with *Streptococcus pneumoniae* D39 (type 2). Subsequently, the different mice groups were treated by intraperitoneal injections of Ac2-26 (1 mg/kg body weight) 2, 8, and 24 h post-infection. The extent of inflammation was analyzed in various brain regions by means of immunohistochemistry and real-time reverse transcription polymerase chain reaction (RT-PCR) 30 h post-infection.

**Results:**

Ac2-26-treated WT mice showed less severe neutrophil infiltration, paralleled by a reduced induction of pro-inflammatory glial cell responses in the hippocampal formation and cortex. While meningitis was ameliorated in Ac2-26-treated *Fpr1*-deficient mice, this protective effect was not observed in *Fpr2*-deficient mice. Irrespective of Ac2-26 treatment, inflammation was more severe in *Fpr2*-deficient compared to *Fpr1*-deficient mice.

**Conclusions:**

In summary, this study demonstrates anti-inflammatory properties of Ac2-26 in a model of bacterial meningitis, which are mediated via FPR2, but not FPR1. Ac2-26 and other FPR2 modulators might be promising targets for the development of novel therapies for *Streptococcus pneumoniae*-induced meningitis.

## Background

Bacterial meningitis, which is characterized by severe inflammation culminating in neuronal damage, is among the top ten causes of infectious disease-related deaths worldwide, with up to half of the survivors left with permanent neurological sequelae. Despite availability of antibiotic therapies, the mortality rate remains high [1]. Most cases of bacterial meningitis begin with host acquisition of a new organism by nasopharyngeal colonization followed by systemic invasion and development of a high-grade bacteremia. The causative pathogens, such as *Neisseria meningitides*, *Streptococcus pneumoniae*, or *Haemophilus influenzae* type B, which are the most common causes of meningitis in infants and adults, can penetrate the central nervous system (CNS) via the blood-brain barrier (BBB). By production and/or release of cytokines within the CNS, meningeal pathogens increase the BBB permeability, triggering the recruitment of peripheral immune cells, among monocytes and neutrophils. There is then an intense subarachnoid space inflammatory response, which leads to many of the pathophysiologic consequences of bacterial meningitis, including cerebral edema and increased intracranial pressure [2].

The main effector cells of the innate immune response within the CNS are the glial cells, in particular microglial cells and astrocytes. Resident microglial cells and astrocytes exert multiple functions including protective and restorative effects in response to CNS infection or injury [3]. For the initiation of a glial cell-driven immune response, astrocytes and microglial cells are equipped with a variety of receptors able to identify an almost infinite antigenic repertoire of broadly defined molecular motifs from pathogens or endogenous molecules. These molecular determinants, so-called pathogen-associated molecular patterns (PAMPs) or danger-associated molecular patterns (DAMP), are conserved molecular structures that are sensed by so called pattern recognition receptors (PRRs). The PRRs comprise multiple receptor families located in both extracellular and intracellular milieus including the toll-like receptors or the G-protein-coupled formyl peptide receptors (FPRs) [4, 5].

The murine *Fpr* gene family has at least six members in contrast to only three in humans. The two most important members are the Fpr1 and Fpr2. *Fpr1* encodes murine Fpr1, which is considered the murine ortholog of human FPR, whereas *Fpr-rs2* (*Fpr2*) encodes a receptor that is most similar to the human formyl peptide receptor-like 1 (FPRL1) or FPR2 [6, 7]. It is well-known that FPRs are expressed by glial cells [6, 8, 9]. Relatively little is known about the functional role of FPR during bacterial meningitis. In a previous study, we used mFPR1 and mFPR2-deficient mice to investigate the effects on inflammation, bacterial growth, and mortality in a mouse model of pneumococcal meningitis. Compared to wild-type mice, mFPR1- or mFPR2-deficient mice showed an increased bacterial burden, increased neutrophil infiltration, and a higher mortality rate. This aggravated disease course was paralleled by a dysregulation of the brain intrinsic inflammatory response [10].

The FPRs are characterized by a broad ligand spectrum. For example, FPRs can be activated by n-formyl peptide from bacterial cell walls (fMLF), by eukaryotic mitochondria [5], by the prion protein PrP106-126 [11], by amyloid beta 1-42 from Alzheimer plaques [12, 13], or by capsule proteins of the human immunodeficiency virus [14]. Besides these predominant pro-inflammatory ligands, FPRs can also be activated by anti-inflammatory molecules such as the endogenous protein annexin A1 or its N-terminal fragment Ac2-26 [15]. With respect to Ac2-26, it has been shown that Ac2-26 inhibits the adhesion and transmigration of leukocytes, thus limiting the intensity and duration of the inflammatory response and supporting the proliferation and immigration of epithelial cells [16, 17]. For Ac2-26 and annexin A1, an anti-inflammatory effect was shown in multiple sclerosis, pneumococcal pneumonia, arthritis, and uveitis, as well as during wound healing [18–20].

In this study, we investigated the effects of the synthetic FPR agonist Ac2-26 in a mouse model of pneumococcal meningitis. We demonstrate a protective effect of Ac2-26 in this model which is mediated via the FPR2 receptor. Ac2-26 treatment resulted in pronounced anti-inflammatory effects, paralleled by an amelioration of astrocyte activation. These findings highlight the potential of members of the FPR family, in particular Ac2-26, as a promising therapeutic option in bacterial meningitis.

## Methods

### Reagents

Ac2-26 (Ac-AMVSEFLKQAWFIENEEQEYVQTVK) was purchased from Bankpeptide Limited (Hefei, China). The peptide was dissolved in phosphate-buffered saline (PBS) buffer. The mice were injected intraperitoneally with Ac2-26 (1 mg/kg body weight) 2, 8, and 24 h after the infection.

### Animals

*Fpr1*^−/−^, *Fpr2*^−*/*−^, and wildtype (WT) mice were kept at the Central Animal Care Facility of the RWTH Aachen University. *Fpr1*^−/−^ mice were a kind gift from Dr. Philip Murphy of the National Institute for Allergy and Infectious Diseases, NIH, Bethesda, MD [21]. The *Fpr2*^−/−^ mice were generated as described previously [22]. Both mouse strains were maintained at a C57BL/6 background. The wild-type (WT) mice were back-crossed on the C57BL/6 J background for at least five generations. The resulting WT were used as control mice. Male mice were used in the experiments to rule out any hormonal effects in the female mice.

### Induction of experimental pneumococcal meningitis

**Fig. 1:**
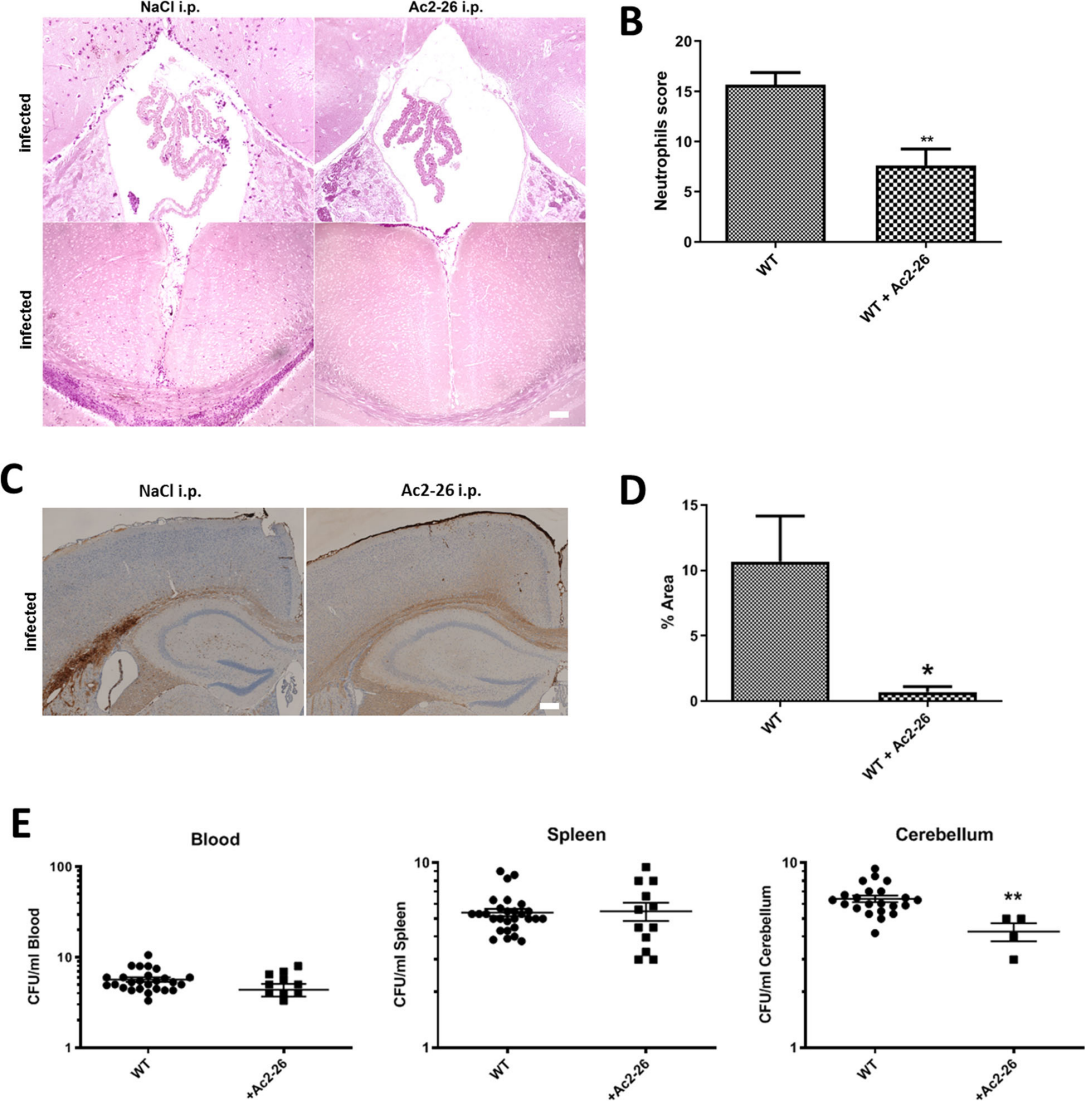
Ac2-26 ameliorates meningeal granulocyte infiltration and bacterial load during pneumococcal meningitis. Wildtype (WT) mice were infected by application of 10^4^ colony-forming units (CFU) of *Streptococcus pneumoniae* D39 type 2 strain into the subarachnoid space with or without Ac2-26 i.p. injection 2, 8, and 24 h after infection. **a** Ac2-26 reduces neutrophil infiltration into the choroid plexus of the third ventricle (left site) and the frontal interhemispheric region (right site), demonstrated by naphthol AS-D chloroacetate esterase (NCAE) reaction. The figures show representative results from one of five to seven mice per group. **b** Quantification of neutrophil granulocyte infiltration using neutrophil score (see “Methods” section for detail; *t* test; **p < 0.01). **c** Coronal brain sections stained with antibodies against neutrophil granulocytes 28 h after infection with *Streptococcus pneumoniae* with or without Ac2-26 i.p. injection 2, 8, and 24 h after infection (scale bar = 100 μm). **d** Densitometric quantification of neutrophil staining was assessed from five mice per experimental group (*t* test; *p < 0.05). **e** Quantification of the bacterial load in the blood, spleen, and cerebellum of infected WT mice with or without Ac2-26 treatment (*n* ≥ 10 each group; Mann-Whitney *U* test; **p = 0.004 for WT vs. WT + Ac2-26)

All in vivo experiments were approved by the Animal Care Committee of the University Hospital of Aachen and by the District Government in Recklinghausen, North Rhine-Westphalia, Germany (84-02.04.2015.A157), and performed according to international ARRIVE guidelines on the use of laboratory mice [23]. The mouse model of pneumococcal meningitis used in this study has been previously described [10, 24, 25]. In brief, 8-week old WT, *Fpr1*^−/−^, and *Fpr2*^−*/*−^ mice were anaesthetized with ketamine (100 mg/kg body weight) and xylazine (20 mg/kg body weight) and infected with the *Streptococcus pneumoniae* D39 (type 2) strain (10^4^ colony-forming units/ml). The bacteria were injected directly through the skull into the subarachnoid space. Control mice received a sterile saline solution. Infected mice developed clinical signs of infection within the first 24 h (see as well Fig. 1). For histology, gene expression studies, and blood analyses, mice were sacrificed within 28 to 30 h post-infection (each group *n* ≥ 5). Unless stated otherwise, three independent experiments were performed. By the end of the experiments, the mice were euthanized and perfused with either 4% formalin or 0.9% NaCl for immunohistochemistry or gene expression studies, respectively. Brain hemispheres were divided in the sagittal plane and used for subsequent analyses. The bacterial burden was analyzed in the spleen and cerebellum as well as in each mouse’s blood sample as described by our group [26].

### Immunohistochemistry

For immunohistochemistry, sections were rehydrated and, if necessary, antigens were unmasked with Tris/EDTA buffer (pH 9.0) or citrate (pH 6.0) heating as previously described [8, 24]. The sections were washed in PBS and incubated overnight at 4 °C with the following primary antibodies diluted in blocking solution (i.e., serum of the species in which the secondary antibody was produced), anti-GFAP (1:75.000; RPCA-GFAP, EnCor, Gainesville, FL, USA), anti-IBA1 (1:10.000; 019-19741, Wako, Neuss, Germany), or anti-neutrophil (1:300; ab2557, Abcam, Berlin, Germany). On the following day, the slides were incubated with biotinylated secondary antibodies (1:50; BA-1000; Biozol, Eching, Germany) for 1 h and, after another washing step in PBS, incubated with a peroxidase-coupled avidin-biotin complex (ABC kit; Vector Laboratories, Peterborough, UK). Finally, the sections were treated with 3,3′-diaminobenzidine (DAKO, Hamburg, Germany) as a peroxidase substrate. After visualization of the antigen-antibody complexes, the slides were counterstained with hematoxylin and covered with DePeX (Serva, Heidelberg Germany).

Stained and processed sections were digitalized using the BZ-9000 microscope from Keyence (Keyence, Neu-Isenburg, Germany). The densities of immunolabeled cells are expressed as cells per square millimeter within the respective region of interest (ROI). To quantify cell/particle density, the stained and processed sections were digitalized using a Nikon ECLIPSE E200 microscope (Nikon Instruments, Düsseldorf, Germany) equipped with a camera (Basler acA1920-40uc) and the software (ManualWSI 2019, Microvisioneer, Germany) to produce scans of the respective sections and regions of interest (ROI). These scans were analyzed using the open source program ViewPoint Online (version 1.0.0.9628, PreciPoint, Freising, Germany). The area of the ROI was manually outlined, and positive cells or particles were counted by an evaluator blinded to the treatment groups. For anti-neutrophil immunohistochemical stains, the staining intensities were quantified by densitometrical analyses. To this end, the open source program ImageJ (version 1.44 h; Java 1.6.0_10, NIH, Bethesda, MD, USA) was used to evaluate staining intensity through semi-automated densitometrical evaluation after threshold setting.The acquired images were converted into gray scale images, and a global thresholding algorithm was used to divide each image into two classes of pixels (black and white; i.e., binary conversion). Global thresholding works by selecting a cutoff value and classifying every pixel lower than that value as one class and every pixel higher than that as another class. Relative staining intensity was then quantified in binary converted images, and the results were determined as the percentage area, with 0% and 100% corresponding to the minimum and maximum stained areas, respectively [26, 27].

### Meningeal inflammation score

The extent of granulocyte infiltration was used as a surrogate marker for meningeal inflammation. Here, the activity of naphthol AS-D chloroacetate esterase (CAE) was used to visualize neutrophil granulocytes (91-C Kit; Sigma-Aldrich, Munich, Germany) according to the manufacturer’s instructions [8, 24]. Granulocyte density was determined 30 h post-infection, in the following ROIs: frontal interhemispheric region, the hippocampal fissure (both sides), three randomly chosen superficial meningeal regions over the entire convexities, and within the third ventricle. For each region, granulocyte density was evaluated in one field of view (× 20 objective), according to the following score: no granulocytes—0; < 10 granulocytes—1; 10 to 50 granulocytes—2; and > 50 granulocytes—3. The sum of the individual regions can therefore reach a maximum value of 21 [25, 28].

### RNA isolation and real-time reverse transcription polymerase chain reaction (RT-PCR) analyses

Following brain perfusion with 0.9% NaCl and brain dissection, RNA was prepared using peqGold Trifast reagent (Peqlab, Erlangen, Germany) as previously described [24]. For cDNA synthesis, Moloney murine leukemia virus (MMLV) reverse transcriptase (Fermentas, Burlington, Canada) and random hexamer primers (Invitrogen, Darmstadt, Germany) were used. Gene expression levels were then determined using SYBR green (Biotool, Houston, USA) real-time PCR and a StepOne Plus apparatus (Applied Biosystems, Darmstadt, Germany). The ΔΔCt method was used for the relative quantification of gene expression levels. Ribosomal protein L13a (*Rpl13a*) was used as internal reference gene. To determine *Gfap* or *Itgam* expression levels, the QuantiTect Primer Assay from Qiagen (QT00101143, QT00156471; Qiagen, Hilden, Germany) was used. Primers used to measure *Rpl13a* [8], tumor necrosis factor-α (*Tnf-α*) [25], interleukin 6 (*IL-6*) [25], chemokine (C-C motif) ligand 3 (*Ccl3*) [29], and C-X-C motif chemokine (*Cxcl10*) [30] were produced by Eurofins MWG Operon (Ebersberg, Germany). The sequences and used annealing temperatures were listed in the cited publications. To exclude contamination of the reagents with either RNA or DNA, appropriate negative controls were performed. Melting curve analyses were used to determine reaction specificity, whereas amplification efficiency was determined with the LinRegPCR software package (version 12.7) [24].

### Statistical analysis

All data are given as the arithmetic means ± SEM. Differences between groups were statistically tested using the software package GraphPad Prism 5 (GraphPad Software Inc., San Diego, CA, USA). The D’Agostino and Pearson test was applied to test for Gaussian distribution of the data. Bacterial load (CFU/ml) was statistically compared with the non-parametric Mann-Whitney *U* test. Real-time RT-PCR data were determined as duplicates. The four groups were statistically compared using the two-way ANOVA followed by Bonferroni’s multiple comparison test. The definite statistical procedure applied for the different analyses is provided in the text and figure legends. *p* values ≤ 0.05 were considered statistically significant. The following symbols are used to indicate the level of significance: *p ≤ 0.05, **p ≤ 0.01, ***p ≤ 0.001, ns = not significant.

## Results

### Ac2-26 reduces bacterial load and meningeal granulocyte invasion during pneumococcal meningitis

Neutrophilic inflammation triggered by *S. pneumoniae* is an important feature of pneumococcal meningitis [25, 26]. Therefore, the two parameters (i) meningeal granulocyte invasion and (ii) bacterial load were determined 28 h after infection with *Streptococcus pneumonia* in infected WT mice. As demonstrated in Fig. 1, intraperitoneal Ac2-26 injection in infected WT mice significantly reduced the extent of neutrophil infiltration (Fig. 1 a and b; *n* = 10 each group; **p < 0.01; *t* test). These results were confirmed by visualizing neutrophil granulocytes by immunohistochemistry. In line with our observation using the enzymatic naphthol AS-D chloroacetate esterase reaction, anti-neutrophil immunohistochemistry showed a strong increase of staining intensities in the hippocampal formation, the corpus callosum, and the cortex of WT mice, whereas in the Ac2-26-treated mice, the staining intensity was significantly lower (Fig. 1 c and d; *n* = 5 each group; *p < 0.05; *t* test).

The observed anti-inflammatory effect of Ac2-26 in *Streptococcus pneumonia*-infected WT mice might principally be due to the amelioration of bacteremia in the periphery or due to a modulation of brain-intrinsic inflammatory cascades. To this end, we determined the bacterial load in the spleen tissue, blood, and CNS. As shown in Fig. 1e and Table 1, in the spleen tissue and blood, the bacterial load was comparable in vehicle- and Ac2-26-treated meningitis mice. In contrast, the bacterial load was significantly lower in the cerebellum of Ac2-26- versus vehicle-treated meningitis mice (*n* ≥ 10 each group; Mann-Whitney *U* test; **p = 0.004 for WT vs. WT + Ac2-26). These results suggest that Ac2-26 ameliorates the course of bacterial meningitis by modulating inflammatory responses within the CNS compartment.

**Table 1:** Bacterial load during pneumococcal meningitis

Bacterial load (log CFU/ml)			
	Blood	Spleen	Cerebellum
WT	5.3 (4.5/6)	5.1 (4.6/5.6)	6.2 (5.6/7)
WT + Ac2-26	4.5 (3.5/6.3)	5 (3.5/7.7)	4.5 (3.3/5)**
*Fpr1*^*−/−*^	8 (6.7/9.9)	7 (7/8.7)^###^	9 (7.1/10.1)^###^
*Fpr1*^*−/−*^ + Ac2-26	6.3 (6/7)	7.2 (6.3/7.5)	5.4 (5/7.5)**
*Fpr2*^*−/−*^	6 (5/7.5)	6 (5/7.2)^#^	6.9 (6/8.7)
*Fpr2*^*−/−*^ + Ac2-26	5.3 (5/5.8)	5.3 (4.9/6.4)	6.2 (4/9)

Data are presented as median (25th/75th percentile). Note significantly increased bacterial loads in the spleen of infected *Fpr1*^*−/−*^ or *Fpr2*^*−*/*−*^ mice and cerebellum of infected *Fpr1*^*−/−*^ mice compared to infected WT mice (*n* ≥ 10 each group; Mann-Whitney *U* test; ^###^*p* = 0.0008 or p = 0.0001 for *Fpr1*^*−/−*^ vs. WT; ^#^*p* = 0.04 for *Fpr2*^*−/−*^ vs. WT), and decreased bacterial loads in the cerebellum of infected WT or *Fpr1*^*−/−*^ mice with Ac2-26 compared to infected WT or *Fpr1*^*−/−*^ mice without Ac2-26 (*n* ≥ 10 each group; Mann-Whitney *U* test; **p = 0.004 for WT vs. WT + Ac2-26; *p* = 0.007 for *Fpr1*^*−/−*^ vs. *Fpr1*^*−/−*^ + Ac2-26) (*CFU*, colony-forming units; WT, wild type)

### Ac2-26 ameliorates pro-inflammatory responses during pneumococcal infection

**Fig. 2:**
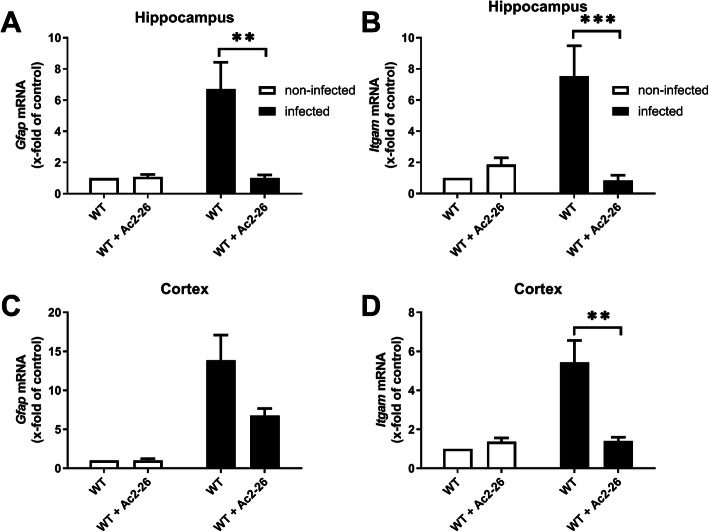
Ac2-26 reduces glial cell marker expression during pneumococcal meningitis. Gene expression was performed 28 h after pneumococcal meningitis. Glial fibrillary acidic protein (*Gfap*; astrocyte marker) (**a** and **c**) and integrin alpha M (*Itgam*; microglia marker) (**b** and **d**) mRNA expression levels were determined in the hippocampal formation and cortex of infected and non-infected WT mice with or without Ac2-26 treatment by real-time RT-PCR (all groups *n* = 5; two-way ANOVA followed by Bonferroni test; **p < 0.01; ***p < 0.001)

The activation of astrocytes and microglial cells leads to the massive production of cytokines and chemokines followed by neutrophil recruitment during the course of bacterial meningitis [31, 32]. In the next step, we were interested whether Ac2-26-treated mice show less severe glial cell reactivity. As surrogate marker for glial cell reactivity, we quantified the expression levels of *Gfap* as astrocytes and *Itgam* as microglial cell markers by real-time RT-PCR in isolated hippocampal and cortical tissues. As shown in Fig. 2a/c, the infection with *Streptococcus pneumoniae* resulted in a robust increase of *Gfap* mRNA expression in the hippocampus (6.7-fold of control) and cortex (13.8-fold of control). Ac2-26 treatment ameliorated *Streptococcus pneumoniae*-induced *Gfap*-expression in WT mice, which was significant for the isolated hippocampus (Fig. 2a; *p* < 0.01; two-way ANOVA followed by Bonferroni test). Comparably, the infection with *Streptococcus pneumoniae* resulted in a robust *Itgam* expression increase in both, the hippocampus (7.5-fold of control) and cortex (5.4-fold of control) of WT mice, and Ac2-26 treatment ameliorated *Streptococcus pneumoniae*-induced *Itgam* expression in WT mice (Fig. 2 b and d; *p* < 0.001 or *p* < 0.01; WT + Ac2-26 vs. WT infected).

**Fig. 3:**
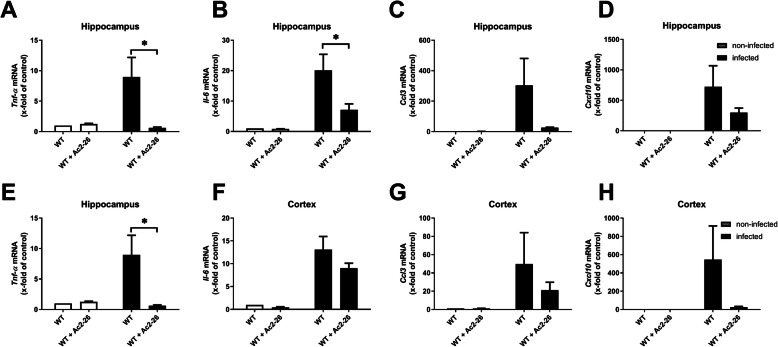
Ac2-26 ameliorates pro-inflammatory responses during pneumococcal meningitis. Twenty-eight hours after subarachnoid infection with *Streptococcus pneumoniae*, mRNA expression of tumor necrosis factor-α (*Tnf-α*) (**a** and **e**), interleukin 6 (*IL-6*) (**b** and **f**), *Ccl3* (**c** and **g**), and *Cxcl10* (**d** and **h**) were determined in the hippocampal formation and cortex of infected and non-infected WT mice with or without Ac2-26 treatment by real-time RT-PCR (all groups *n* = 5; two-way ANOVA followed by Bonferroni test; *p < 0.05; **p < 0.01)

Next, gene expression levels of the pro-inflammatory cytokines *TNF-α* or *IL-6* as major mediators of the inflammatory reaction during pneumococcal meningitis were determined in the hippocampal formation as well as cortex 28 h after infection. The infection resulted in an increase of *TNF-α* or *IL-6* mRNA expression in the hippocampus and cortex of infected WT mice. Ac2-26 treatment significantly reduced *TNF-α* mRNA expression in both brain regions (Fig. 3 a and e; *p* < 0.05; two-way ANOVA followed by Bonferroni test), whereas *IL-6* mRNA expression was significantly reduced in the hippocampus (Fig. 3b; *p* < 0.05; two-way ANOVA followed by Bonferroni test), and by trend in the cortex.

Besides cytokines, chemokines are also an important part of the host innate immune response. Therefore, the gene expression levels of *Ccl3*, which causes activation of polymorphonuclear leukocytes [33] and *Cxcl10* as a pro-inflammatory chemotactic agent for monocytes/macrophages, T cells, NK cells, and dendritic cells [33], were quantified in the same tissue samples. In infected WT mice, *Streptococcus pneumoniae*-induced meningitis lead to a robust and significant increase of *Ccl3* and *Cxcl10* expression levels. *Ccl3* and *Cxcl10* expression induction was by trend less severe in Ac2-26-treated meningitis mice (Fig. 3 c, d, g, and h; *p* > 0.05; two-way ANOVA followed by Bonferroni test).

### Ac2-26 reduces astrocyte activation after pneumococcal meningitis

**Fig. 4:**
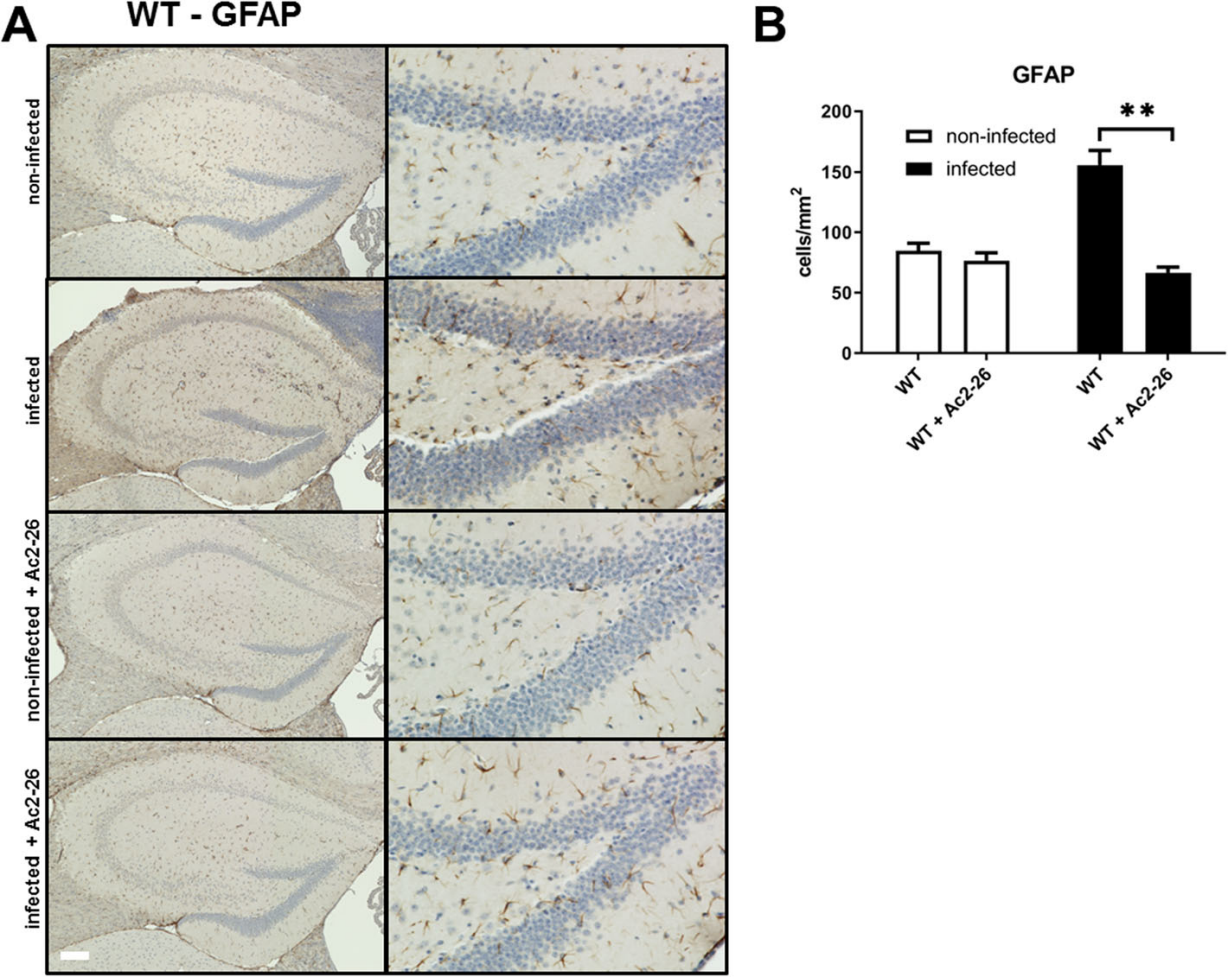
Ac2-26 reduces density of astrocytes during pneumococcal meningitis*.* **a** Coronal brain sections were stained with antibodies against glial fibrillary acidic protein (GFAP) to identify activated astrocytes 28 h after infection with *Streptococcus pneumoniae*. The right column shows the dentate gyrus in higher magnification (scale bar = 100 μm). **b** GFAP immunoreactive cells were quantified, and the results are shown as cells per mm^2^ area of dentate gyrus (all groups *n* = 5; two-way ANOVA followed by Bonferroni test; **p < 0.01 compared to infected WT mice)

**Fig. 5:**
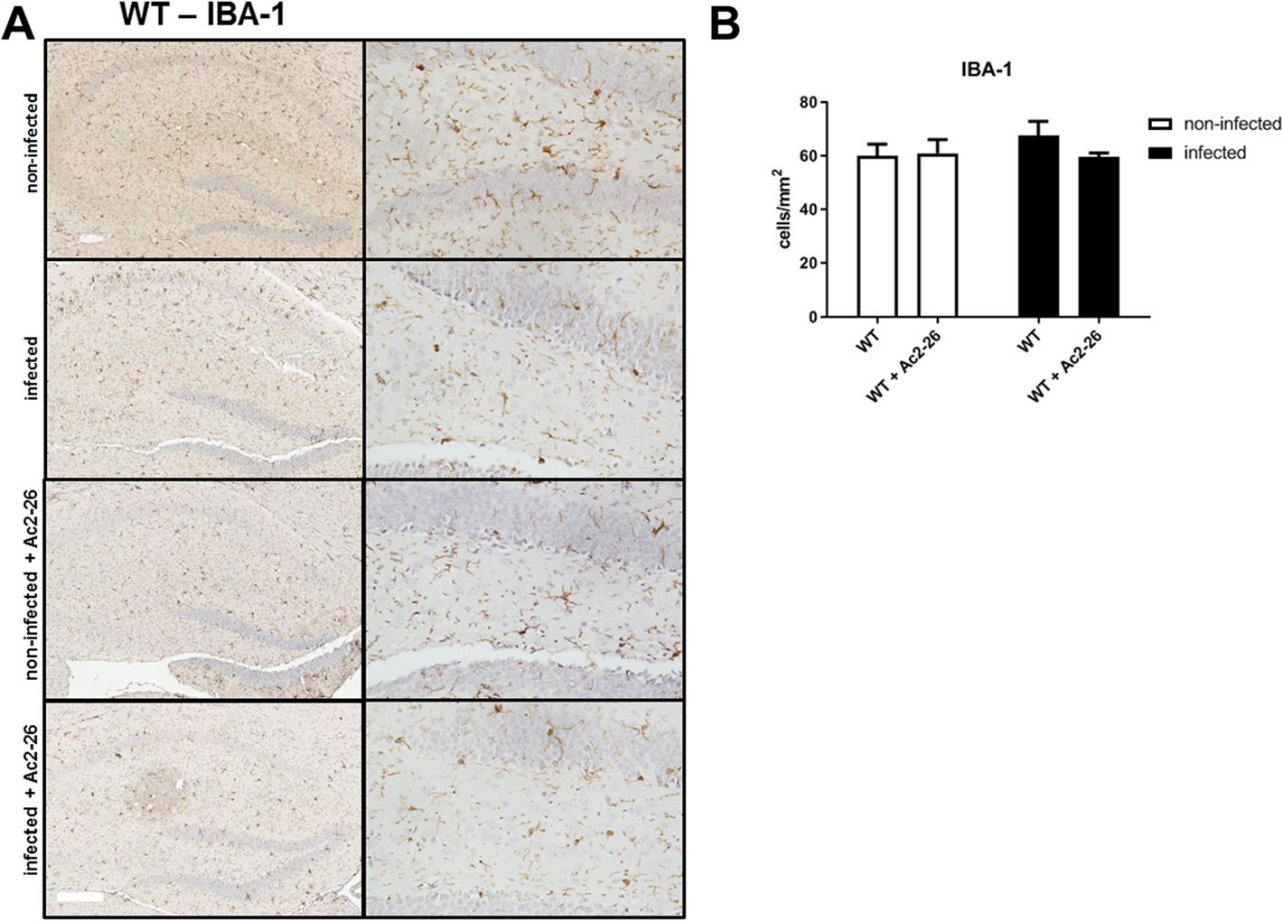
Ac2-26 does not change density of microglial cells during pneumococcal meningitis. **a** Coronal brain sections were stained with antibodies against ionized calcium-binding adaptor molecule 1 (IBA-1) to identify activated microglial cells 28 h after infection with *Streptococcus pneumoniae*. The right column shows the dentate gyrus in higher magnification (scale bar = 100 μm). **b** IBA-1 immunoreactive cells were quantified, and the results are shown as cells per mm^2^ area of dentate gyrus (all groups *n* = 5; two-way ANOVA followed by Bonferroni test; *p* > 0.05 compared to infected WT mice)

Results of our gene expression studies suggest that astrocyte or glial cell activation during *Streptococcus pneumoniae*-induced meningitis is ameliorated by Ac2-26 treatment. To verify this finding on the protein and cellular levels, we used anti-GFAP or anti-IBA-1 immunohistochemistry to assess the density of astrocytes or microglial cells in the entire hippocampal formation. In line with our results on the mRNA level, the density of GFAP-expressing cells increased in meningitis-induced mice, and this increase was ameliorated by Ac2-26 (Fig. 4b; *p* < 0.01; two-way ANOVA followed by Bonferroni test). For IBA-1-positive cells (marker for microglial cells), only a very weak increase of the density was observed after infection in WT mice (Fig. 5 a and b). The injection of Ac2-26 did not result in significant differences compared to the WT mice.

### Protective Ac2-26 effects are mediated by FPR2

**Fig. 6:**
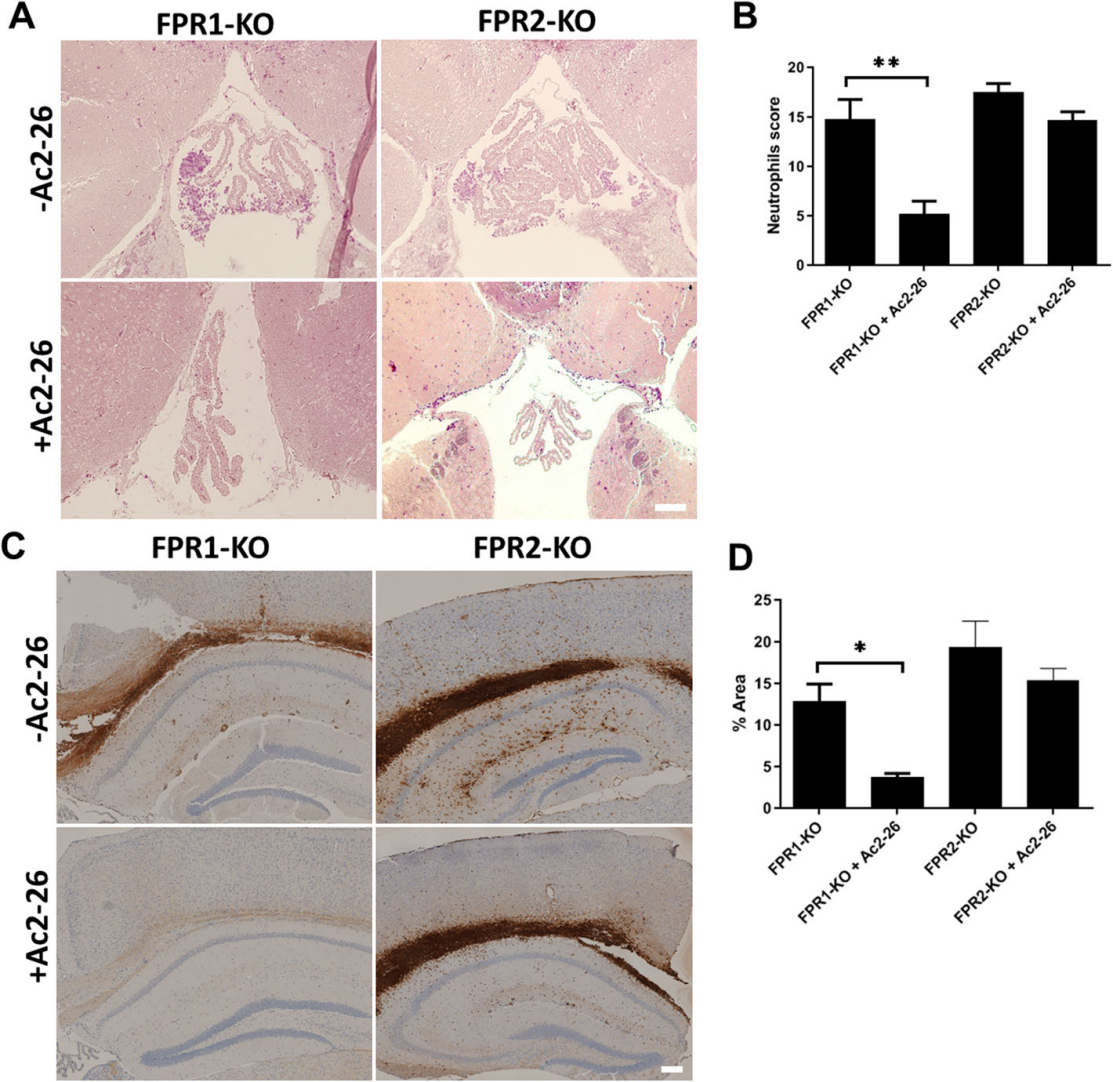
Ac2-26 protective effects are mediated via FPR2. **a** Detection of neutrophil granulocytes by naphthol AS-D chloroacetate esterase (NCAE) reaction in the third ventricle demonstrates less severe neutrophil infiltration in Ac2-26-treated *Fpr1*^*−/−*^, but not *Fpr2*^*−/−*^ mice 28 h post-infection. The figures show representative results from one of five to seven mice per group. **b** Quantification of neutrophil granulocyte infiltration using neutrophils score (see “Methods” section for detail; one-way ANOVA followed by Bonferroni test; **p < 0.01). **c** Coronal brain sections of *Fpr1*^*−/−*^ or *Fpr2*^*−/−*^ mice were stained with antibodies against neutrophil granulocytes 28 h after infection with *Streptococcus pneumoniae*, with or without Ac2-26 i.p. injection (scale bar = 100 μm). **d** Densitometric quantification of neutrophil staining was assessed from five mice per experimental group (*t* test; *p < 0.05)

It has been demonstrated that FPR2, also known as the lipoxin A4 receptor (ALX), is a cell surface receptor for annexin A1 [34, 35]. We, thus, speculated that the N-terminal fragment of annexin A1 (i.e., Ac2-26) mediates its protective effect as well through the FPR2. To investigate this aspect, we used *Fpr1*^*−/−*^ and *Fpr2*^*−/−*^ mice with or without Ac2-26 injection to analyze the meningeal granulocyte invasion and bacterial load 28 h after infection. As shown in Fig. 6, the infection of *Fpr1*^*−/−*^ or *Fpr2*^*−/−*^ mice resulted in a comparable extent of neutrophil infiltration as compared to infected WT mice (compare to Fig. 1). Thus, *Fpr1*^*−/−*^ and *Fpr2*^*−/−*^ mice were equally susceptible to the infection compared to their WT counterparts. Of note, while Ac2-26 was still able to reduce neutrophil infiltration in *Fpr1*^*−/−*^ mice, such a protective effect was not evident in *Fpr2*^*−/−*^ mice (*n* = 10 each group; **p < 0.01; one-way ANOVA followed by Bonferroni test). The observed results were confirmed by immunohistochemistry. In line with our observation using the enzymatic naphthol AS-D chloroacetate esterase reaction, anti-neutrophil immunohistochemistry showed a comparable increase of staining intensities in the hippocampal formation, the corpus callosum, and the cortex of WT mice. The Ac2-26 treatment reduced the staining intensity in *Fpr1*^*−/−*^ mice significantly whereas in *Fpr2*^*−/−*^ mice no protective effect could be observed (Fig. 6 c and d; *n* = 5 each group; *p < 0.05; *t* test).

As demonstrated in Table 1, the reduced neutrophil infiltration in Ac2-26-treated WT and *Fpr1*^*−/−*^ mice was paralleled by a lower bacterial load in the cerebellum (6.2 vs. 4.5 in WT mice; 9 vs. 5.4 in *Fpr1*^*−/−*^ mice). In contrast, the cerebellar bacterial load was not reduced in Ac2-26-treated *Fpr2*^*−/−*^ mice (6.9 vs. 6.2).

### Ac2-26 reduces astrocyte responses in *Fpr1*^*−/−*^ but not *Fpr2*^*−/−*^ mice

**Fig. 7:**
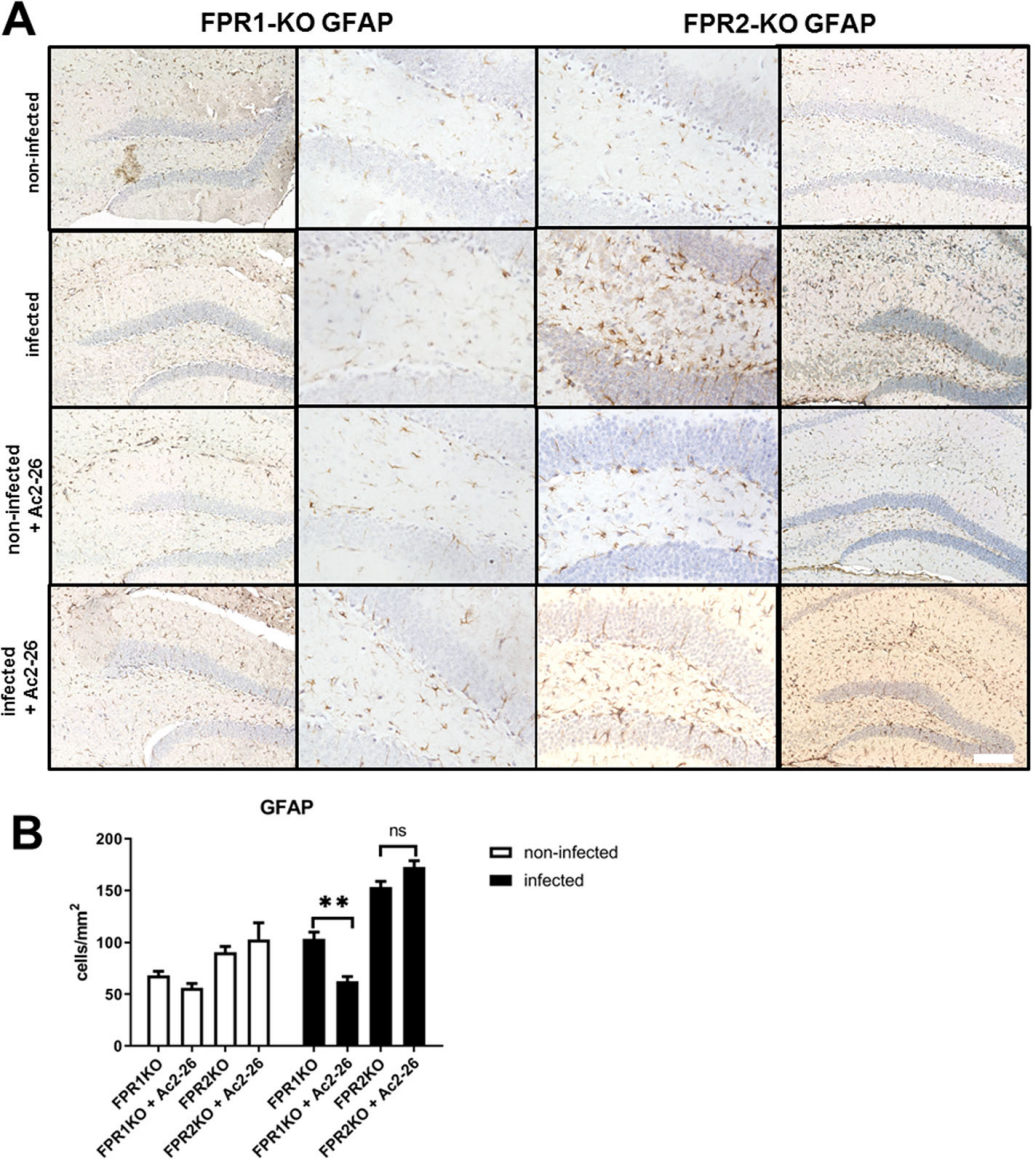
Ac2-26 ameliorates reactive astrogliosis in Fpr1- but not Fpr2-deficient mice. **a** Coronal brain sections stained with antibodies against glial fibrillary acidic protein (GFAP) to identify activated astrocytes 28 h after infection with *Streptococcus pneumoniae* (scale bar = 100 μm). **b** GFAP immunoreactive cells were quantified, and the results are shown as cells per mm^2^ area of dentate gyrus (all groups *n* = 5; two-way ANOVA followed by Bonferroni test; **p < 0.01)

**Fig. 8:**
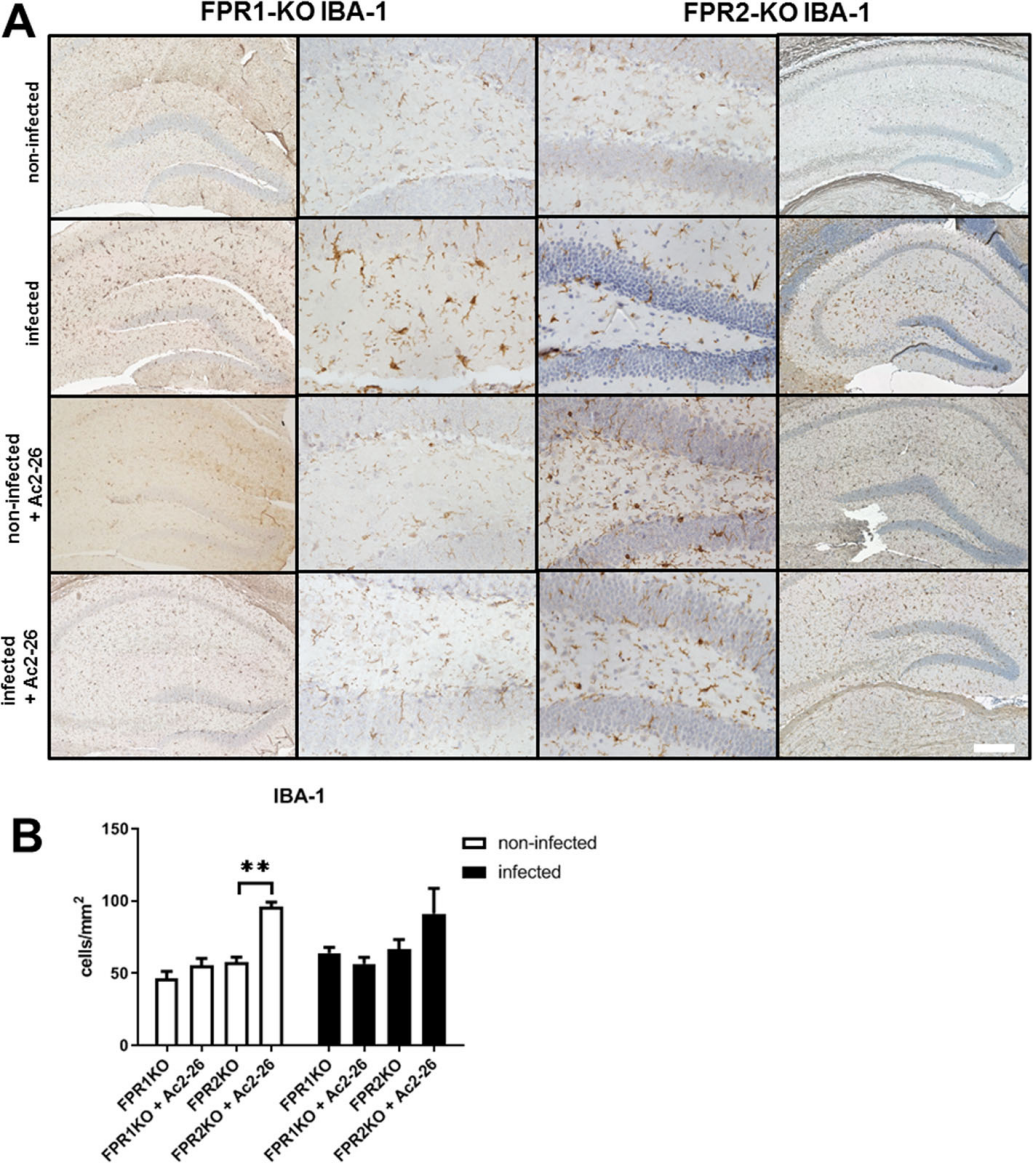
Ac2-26 did not change density of microglial cells during pneumococcal meningitis in Fpr1- or Fpr2-deficient mice. **a** Coronal brain sections were stained with antibodies against ionized calcium-binding adaptor molecule 1 (IBA-1) to identify resting and activated microglial cells 28 h after infection with *Streptococcus pneumoniae* (scale bar = 100 μm). **b** IBA-1 immunoreactive cells were quantified, and the results are shown as cells per mm^2^ area of dentate gyrus (all groups *n* = 5; two-way ANOVA followed by Bonferroni test; **p < 0.01)

Finally, we were interested whether the meningitis-induced astrocyte and microglia response are ameliorated in Ac2-26-treated *Fpr1*^*−/−*^ and *Fpr2*^*−/−*^ mice. To this end, we investigated the densities of GFAP^+^ cells using immunohistochemistry in *Fpr1*^*−/−*^ or *Fpr2*^*−/−*^ mice with or without Ac2-26 treatment. As shown in Fig. 7a, the infection with *Streptococcus pneumoniae* resulted in an increased GFAP^+^ cell density in *Fpr1*^*−/−*^ and *Fpr2*^*−/−*^ mice. The meningitis-induced increase in GFAP^+^ cell densities was ameliorated in *Fpr1*^*−/−*^ but not *Fpr2*^*−/−*^ mice (*p* < 0.01; two-way ANOVA followed by Bonferroni test). In line with our findings in WT mice, anti-IBA1 staining intensities were not found to be increased in infected mice (Fig. 8a). Of note, the treatment with Ac2-26 increased anti-IBA1 staining intensities in both, non-infected and infected *Fpr2*^*−/−*^ mice (Fig. 8b; *p* < 0.01; for *Fpr2*^*−/−*^ + Ac2-26 vs. *Fpr2*^*−/−*^ non-infected).

## Discussion

**Fig. 9:**
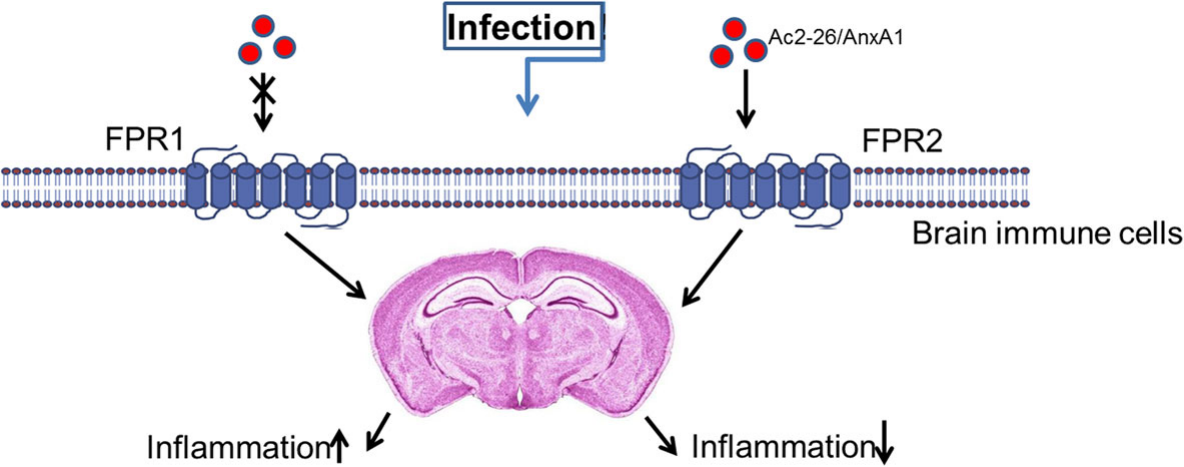
Schematic model of Ac2-26/AnxA1 peptide mechanism of action in the pneumococcal meningitis. Ac2-26 mediated about FPR2 expressed by the brain immune cells the anti-inflammatory response after pneumococcal infection. This results in an improvement in the inflammatory reaction. The activation of the FPR1 during the infection tends rather to an increase of the inflammatory response

In this study, WT mice were infected with a high inoculum of *Streptococcus pneumoniae*, treated with Ac2-26 directly after the infection and euthanized 28 h later. This inoculum of *Streptococcus pneumoniae* resulted in marked leukocyte recruitment, especially neutrophils, in the CNS of infected mice paralleled by pronounced astrocyte activation. Treatment with Ac2-26 decreased leukocyte recruitment and astrocyte reactivity. Pneumococcal infection as well increased the mRNA expression levels of pro-inflammatory cytokines and chemokines, including *Tnf-α*, *Il-6*, *Ccl3*, and *Cxcl10*, which were equally reduced after treatment with Ac2-26. Most importantly, the protective Ac2-26-mediated effects were absent in *Fpr2*^*−/−*^ mice (see Fig. 9 for schematic conclusion).

Bacterial meningitis is associated with a strong neuroinflammatory response and despite highly specialized treatments may lead to permanent neurological disability or even death [1]. In the present study, we investigated the effects of the anti-inflammatory FPR ligand Ac2-26 on the inflammatory response in a murine pneumococcal meningitis model. Ac2-26 is the bioactive N-terminal fragment of the endogenous protein annexin A1 [15]. Annexin A1 is a glucocorticoid-regulated phospholipid-binding protein that suppresses both, the innate and adaptive immune response by for example inhibition of adhesion and transmigration of leukocytes. It limits the intensity and duration of the pro-inflammatory response and supports the proliferation and migration of epithelial cells as an endogen repair mechanism [36]. The anti-inflammatory properties of Ac2-26 were shown in inflammatory/autoimmune disease models like rheumatoid arthritis, asthma bronchiale, or ocular inflammation [37–39].

In this study, we were able to demonstrate that the treatment of pneumococcal-infected WT mice with Ac2-26 results in a reduction of neutrophil granulocyte recruitment and bacterial load, paralleled by a reduced glial cell activation and pro-inflammatory cytokine expression induction. An attenuation of neutrophil infiltration by Ac2-26 was as well demonstrated in a pre-clinical model of acute lung injury, myocardial infarction, and acute gout [40–42]. On the mechanistic level, Dalli et al. showed that Ac2-26 exerts chemokinetic effects on human neutrophils, and Machado et al. revealed that annexin A1/Ac2-26 is a physiological modulator of neutrophil maturation and recirculation [43, 44]. Of note, results from Galvao and colleagues suggest that Ac2-26 might induce neutrophil apoptosis [42]. Whether such an effect is operant in our applied meningitis model remains to be verified in future studies.

Lower neutrophil counts in the CNS of Ac2-26-treated meningitis mice might principally be due to a stimulation of bacterial phagocytosis by Ac2-26 in the periphery [19]. To verify or falsify this assumption, we measured in parallel the bacterial load in the peripheral circulation (i.e., blood and spleen) as well as in the CNS (i.e., cerebellum). As demonstrated in Fig. 1e, Ac2-26 treatment did not affect the bacterial burden in the periphery but in the CNS. This finding strongly suggests that Ac2-26 modulates the CNS-intrinsic inflammatory response, by modulating glial reactivity. Although not addressed in this study, microglial cells have been shown to be able to phagocytose neutrophil cells [45]. Future studies have to address whether the neutrophil phagocytosis by microglial cells takes place during bacterial meningitis, and whether such a presumably protective effect is mediated by FPR in general, and by FPR2 in particular.

Astrocytes are the most abundant glial cell type within the CNS and are essential for brain homeostasis. As immune-competent cells, they secrete a plethora of cytokines and chemokines, provide metabolites and growth factors to neurons, support synapse formation and plasticity, and participate in blood-brain barrier maintenance and permeability [46, 47]. Furthermore, they are responsible for pathogen recognition and immune cell recruitment during adaptive immune responses [32, 48]. It has been demonstrated that FPRs are expressed by astroglial cells [9, 49]. We, therefore, asked whether the protective effect of Ac2-26 might be mediated via an interaction with astrocytes. As demonstrated in Figs. 2 or 3, the astroglial cell responses, as determined by anti-GFAP immunohistochemistry and *Gfap* mRNA quantifications, revealed strong GFAP expression induction in meningitis mice, which was robustly ameliorated by Ac2-26 treatment. While these results convincingly show that astrocyte activation is less severe in Ac2-26-treated mice, this does not necessarily mean that a direct, astrocyte-mediated protective effect of Ac2-26 is operant. Conditional *Fpr* knock-out mice would be required to investigate this important aspect more in detail. The protective Ac2-26-mediated effects were as well observed in *Fpr1*^−/−^ but not *Fpr2*^−/−^ mice. These results strongly suggest that the immunomodulatory actions of the AnxA1 peptide Ac2-26 on pneumococcal infection were FPR2-dependent. Our previous works demonstrated the importance of microglial cells within the inflammatory process during pneumococcal meningitis [10, 50]. In the present study, the evaluation of microglial cells did not reveal significant differences for microglial cell density after infection or with Ac2-26 injection. However, the mRNA analysis of a microglial cell activation marker *Itgam* indicates an increase of activation whereas Ac2-26 injection decreased the cell activation.

Several studies demonstrated that *Fpr1*- and/or *Fpr2*-deficient mice are protected from neuronal pathologies, among studies to experimental demyelination or lipopolysaccharide-induced brain inflammation [8, 51, 52]. In a recent study, we were able to demonstrate that lack of either FPR1 or FPR2 leads to more severe inflammation and higher mortality in *Streptococcus pneumoniae*-infected mice [10]. In the present study, in all investigated genotypes (i.e., WT, *Fpr1*^−*/*−^, or *Fpr2*^−*/*−^) the intensity of neutrophil recruitment into the CNS was comparable (compare Figs. 1 and 6). In contrast, bacterial loads were higher in the blood, spleen, and CNS of *Fpr1*^−*/*−^ versus WT and *Fpr2*^−*/*−^-infected mice (see Table 1). Of note, the results of the present study are not directly comparable with our previous results. In the current study, mice were sacrificed 28 h after infection, whereas in our previous study, mice were sacrificed at a more advanced disease stage (i.e., 48 h post-infection). Beyond, mortality measurements were done with antibiotic treatment. It, thus, might be that FPR deficiency becomes clinically apparent during advanced disease stages. Our finding that the bacterial loads are higher in the blood, spleen, and CNS of *Fpr1*^−*/*−^ versus WT and *Fpr2*^−*/*−^ infected mice is consistent with our previous finding [10] and is in support for a reduced survival in *Fpr1*^−*/*−^ mice.

In a recent study, it has been shown that Ac2-26 reduces the inflammatory response and bacterial loads after pneumococcal pneumonia and that this protective effect is mediated via FPR2 [19]. Interestingly, in that study, it was observed that FPR2-deficiency results in higher levels of the pro-inflammatory cytokines compared to WT littermates [19]. In our study, we observed higher *Tnfα*, *IL-6*, *Ccl3*, and *Cxcl10* mRNA expression levels in infected *Fpr2*^−*/*−^ compared to WT mice (data not shown). This might be explained by the fact that FPR2 also binds and is activated by other anti-inflammatory mediators including lipoxin A4 or resolvin D1 [35].

It appears that FPR2 mainly mediates anti-inflammatory responses (Fig. 9). Dufton et al. documented an increase in the inflammatory response for FPR2-deficient mice in a serum-induced arthritis model [53]. Furthermore, activation of the FPR2 axis appears to attenuate disease development and/or progression in a murine influenza A virus infection model, orchestrates resolution of mast cell inflammation, or modulates anti-inflammatory responses in the mouse submandibular gland [54–56]. Our own previous works in a mouse model of pneumococcal meningitis and liver injury after lipopolysaccharide inflammation as well suggest an anti-inflammatory role for FPR2 [10, 57]. Of note, the observed potentiation of the cytokine and chemokine expression induction in the absence of FPR2 did not result in an increased neutrophil recruitment. Neuronal and synaptic integrity might well be impaired, and more detailed studies are now needed to investigate neuronal and functional consequences of FPR modulation.

## Conclusion

In summary, we provide strong evidence that Ac2-26 and other FPR2 ligands might be a therapeutic option for the treatment of bacterial infections in general, particularly for bacterial meningitis. Over the past several decades, the incidence of bacterial meningitis in children has decreased but there remains a significant burden of disease in adults, with a mortality of up to 30%. Even with appropriate antimicrobial therapies, mortality is high and so attention has recently focused on adjunctive therapies. Any further improvements in outcome are likely to come from either modulation of the host response or novel approaches to therapy, rather than new antibiotics. Future studies have to show whether these first promising results presented in this paper show effectiveness as well in other models of experimental meningitis and have a good safety profile in humans.

## Data Availability

The datasets used and/or analyzed during the current study are available from the corresponding author on reasonable request.

## Abbreviations

BBB: Blood-brain barrier
CNS: Central nervous system
FPR: Formyl peptide receptor
GFAP: Glial fibrillary acidic protein
Iba-1: Ionized calcium-binding adaptor molecule 1
Itgam: Integrin alpha M
KO: Knock out
WT: Wildtype
